# Inflammatory proteins in infected bone tissue – An explorative porcine study

**DOI:** 10.1016/j.bonr.2020.100292

**Published:** 2020-06-26

**Authors:** Mats Bue, Natasja Leth Bergholt, Louise Kruse Jensen, Henrik Elvang Jensen, Kjeld Søballe, Maiken Stilling, Pelle Hanberg

**Affiliations:** a Aarhus Microdialysis Research Group, Orthopaedic Research Unit, Aarhus University Hospital, Aarhus, Denmark; b Department of Clinical Medicine, Aarhus University, Aarhus N, Denmark; c Department of Orthopaedic Surgery, Aarhus University Hospital, Aarhus, Denmark; d Department of Veterinary and Animal Sciences, University of Copenhagen, Denmark; e Department of Orthopaedic Surgery, Horsens Regional Hospital, Horsens, Denmark

**Keywords:** Inflammatory proteins in infected bone tissue, Fluid space of bone, Microdialysis, Proximity extension assay, Osteomyelitis porcine model

## Abstract

**Objective:**

To explore the in situ inflammatory proteins in the local extracellular fluid of infected bone tissue.

**Material and methods:**

Seven pigs went through a two-step surgery performing a traumatically implant-associated *Staphylococcus aureus* osteomyelitis in the proximal tibia. Five days later, microdialysis catheters (membrane cut off: 20 kDa) were placed in the implant cavity, infected and healthy cancellous bone, and infected and healthy subcutaneous tissue. Plasma samples were collected simultaneously. We employed an antibody-based proximity extension assay (Olink Inflammatory panel) for the measurement of inflammatory molecules within plasma and extracellular fluid of the investigated tissue compartments.

**Results:**

A higher normalized protein expression in the infected bone tissue in comparison to healthy bone tissue was identified for proteins associated with angiogenesis and bone remodeling: OPG, TGFα, MCP-1, VEGFA, and uPA. Moreover, a parallel detectability of the systemic range of cytokines and chemokines as from the investigated local tissue compartments was demonstrated, indicating the same occurrence of proteins in the local environment as within plasma.

**Conclusion:**

An angiogenic and osteogenic inflammatory protein composition within the extracellular fluid of infected bone tissue was described. The findings support the current histopathological knowledge and, therefore, microdialysis may represent a valid method for sampling of material for protein investigation of the in vivo inflammatory composition within the extracellular environment in infected bone tissue.

## 1 Introduction

Bacterial bone infections induce a massive inflammatory response. However, the inflammatory composition in bone tissue emerged from bacterial infections and its impact on the final healing outcome is limited and the current knowledge is primarily based on in-vitro studies. Recently, it has been demonstrated that a bacterial induced inflammatory response was associated with reduced antibiotic bone penetration in an acute implant-associated osteomyelitis porcine model (Jensen et al., 2017b). Within the suppurative bone cavities, the histopathological changes comprised of extensive inflammatory processes characterized by the presence of neutrophils, macrophages, osteoclasts, activated fibroblasts, angiogenesis, oedema, and necrotic bone tissue (Morawietz et al., 2006; Jensen et al., 2017b). Morphologically, these changes are well described, whereas the in vivo biochemistry behind the inflammatory response is poorly investigated (Morawietz et al., 2006; Krenn et al., 2014; Jensen et al., 2017a; Jensen et al., 2017b). The primary reason for this is the lack of a suitable method to assess the in situ inflammatory processes through biochemical reactions. Microdialysis, as a sampling method, has the potential to generate valuable in vivo information regarding the inflammatory processes. Microdialysis is advantaged by dynamic sampling of molecules from the extracellular space and allows for simultaneous sampling from multiple tissues (Muller, 2002; Joukhadar and Muller, 2005). Specifically for bone tissue, microdialysis has until now mainly been applied for the assessment of metabolism and antibiotics (Bogehoj et al., 2011; Lorenzen et al., 2013; Tottrup et al., 2016; Bue et al., 2018b; Hanberg et al., 2019; Tottrup et al., 2019). Antibody-based proximity extension assay (PEA) is a specific and sensitive technique requiring only a small amount of the target analyte within the sample in order to perform precision-, identification- and biomarker protein analysis (Lundberg et al., 2011). The present study aimed to assess the feasibility of applying microdialysis and PEA as a tool for exploring inflammatory molecules in the extracellular fluid of infected bone tissue. Furthermore, it was investigated if local tissue inflammatory proteins were reflected in plasma samples.

## 2 Materials and methods

The study was approved by the Danish Working Environment Authority and The Danish Animal Experiments Inspectorate, and was carried out in accordance with existing laws (license No. 2013/15-2934-00946). All biochemical analyses were performed at BioXpedia A/S, Aarhus, Denmark. The study was performed on a previously described porcine osteomyelitis model (Jensen et al., 2017a). The pigs were included in a pharmacokinetic study of the vancomycin penetration into soft tissue, healthy bone tissue, infected bone tissue and infected implant cavities (Bue et al., 2018a).

### 2.1 Study procedures

Seven female pigs (Danish Landrace breed, weighing from 75 to 86 kg) were included in the study. All pigs went through two surgeries: day 0 and 5.

On day 0, a traumatically implant-associated *Staphylococcus aureus* osteomyelitis was induced in the proximal metaphysis of the right tibia, parallel to and approximately 10 mm distal to the epiphyseal line. A concentration of 10^4^ colony-forming units of a beta-hemolytic *Staphylococcus aureus* strain S54F9 of spa type t1333 (spaserver.ridom.de) in a 10-μL saline solution was inoculated in the cavity (ø: 4 mm, depth 25 mm) with an implant of a 20 × 2 mm Kirschner wire (Johansen et al., 2011; Aalbaek et al., 2015). Fluoroscopic and CT-overview of surgical and sampling sites can be found elsewhere (Bue et al., 2018a).

On day 5, all pigs had developed subcutaneous abscesses adjacent to the implant cavity. In the infected leg, microdialysis catheters were placed in the implant cavity, in a drill hole (ø: 2 mm, depth 25 mm) approximately 8 mm parallel to the implant cavity and 10 mm distal to the epiphyseal line (simulating infected cancellous bone), and in the subcutaneous tissue parallel to and 10 mm distal to the skin incision (simulating infected subcutaneous tissue). In the healthy left tibia, microdialysis catheters were placed identically in a cancellous drill hole and in subcutaneous tissue simulating healthy tissue. The following membrane lengths of the microdialysis catheters were used: implant cavity (20 mm), healthy and infected cancellous bone (20 mm), healthy and infected subcutaneous tissue (30 mm). Correct location of the bone catheters was assessed by fluoroscopy. A more profound description of the surgical procedures can be found elsewhere (Tottrup et al., 2016; Bue et al., 2018a).

One-thousand milligrams of vancomycin was given intravenously and the first (time interval: 0–40 min) and last (time interval: 420–480 min) dialysate in a 8-h sampling period were collected from all compartments from all pigs. Venous blood samples were drawn from a central venous catheter in the middle of every dialysate sampling interval. The dialysates were instantly stored at −80 **°**C until analysis. Venous blood samples were stored at 5 °C for a maximum of 24 h before being centrifuged at 3000*g* for 10 min. Plasma aliquots were then stored at −80 °C until analysis. The pharmacokinetic vancomycin parameters has previously been published (Bue et al., 2018a).

Induction of anesthesia on day 0 and 5 and postoperative care was performed in standardized manner (Tottrup et al., 2016). During the surgical procedures and the entire sampling period (day 5), the pigs were kept under general anesthesia using a combination of fentanyl (0.35–0.5 mg/h, continuous infusion) and propofol (500–600 mg/h, continuous infusion). Core temperature and arterial pH was monitored throughout the study and kept in the range of 36.2–39.1 °C and 7.36–7.47, respectively. Following collection of the last sample, the pigs were euthanized using pentobarbital given intravenously.

### 2.2 Microdialysis

Briefly, microdialysis is a catheter-based method allowing for continuous sampling of molecules from the extracellular fluid in the tissue of interest by means of a semipermeable membrane at the tip of the microdialysis catheter (Muller, 2002; Joukhadar and Muller, 2005). The solution that passes through the catheter can be sampled in small vials for subsequent analysis. The microdialysis setup comprised of CMA 107 precision pumps (μ-Dialysis AB, Stockholm, Sweden) and CMA 70 catheters (molecular cut-off 20 kDa). All the microdialysis catheters were perfused with 0.9% NaCl at a perfusion rate of 1 μL/min throughout the sampling periods. Given the continuous perfusion of the microdialysis system, complete concentration equilibrium across the semipermeable membrane will never occur. Thus, the concentration in the dialysate represents only a fraction of the absolute tissue concentration, expressed as relative recovery. A detailed description of microdialysis can be found elsewhere (Muller, 2002; Joukhadar and Muller, 2005; Hanberg et al., 2020).

### 2.3 Proximity extension assay

The first and last sample in the 8-h sampling period from all compartments from all pigs were used to estimate the local concentrations of the protein profiles comprised in the Olink Inflammatory panel (Olink proteomics) using a human PEA on a Fluidigm BioMark HD realtime polymerase chain reaction (PCR) platform. In brief, 1 μL sample was used for PEA technology: an oligonucleotide-labeled antibody probe was paired to the target protein and brought the oligos in proximity for hybridization. A DNA polymerase was added for amplification and subsequently quantification was performed using microfluidic real-time quantitative PCR (96.96 Dynamic Array™ Integrated Fluidic Circuit (IFC) Fluidigm BioMark) (Lundberg et al., 2011; Assarsson et al., 2014; Greenwood et al., 2015). Three replicates of negative control and three replicates of interplate controls were run together with the samples. Negative controls were used to determine background levels and calculate assay specific limit of detection (LOD). Interplate controls were used for calculation of normalized protein expression (NPX). In addition, each assay contained four internal controls to monitor all steps from incubation to detection(Assarsson et al., 2014). The inflammatory panel allowed for analysis of 92 inflammation-related protein biomarkers across 96 samples simultaneously. A comprehensive list of all protein markers can be found in Supplementary Fig. 1. Data are presented as arbitrary log2-transformed units NPX. Higher NPX values correspond to a higher protein expression. Public-access bioinformatic databases including UniProt, Gene Ontology, KEGG pathways, and STRING were used for the interpretation of the biological relevance of the differently expressed proteins.

### 2.4 Statistics

An explorative cut-off was created for the analysis of immunological impact quantified in microdialysates and plasma specimens. Plasma proteins with a data measured frequency below <80% were excluded from statistical tests and only used descriptive. Proteins with higher measured frequency in microdialysates in comparison to plasma were included. In order to identify possible outliers, and to evaluate consistency of the data, principal component analyses (PCAs) were performed and visualized. PCA was performed for all samples grouped by different variables of interest in R version 3.6.1. PCA plots were illustrated as scatterplots with percentage of variability. All NPX data were checked for normality using histogram and QQ plots. All data are presented as mean ± SE and differences between groups where considered significant when *p* < .05*. One-way ANOVA was performed on arbitrary log-2 scaled NPX values for microdialysates and plasma comparison, and for healthy bone and infected bone comparison. The statistical analyses were performed using STATA statistical Software: release 13.1 (StataCorp LP, Collage Station, TX, USA).

### 2.5 Inter-group assessments

PEA was used for explorative assessment of the inflammatory protein composition differences in the following three collations; (1) local (all microdialysates) vs systemtic (plasma), (2) healthy vs infected recipients (both subcutaneous tissue and bone samples from healthy and infected leg were included), and (3) healthy vs infected bone (infected bone represents the cancellous bone compartment plus the implant cavity as measurability was similar within the two compartments).

**Fig. 1:**
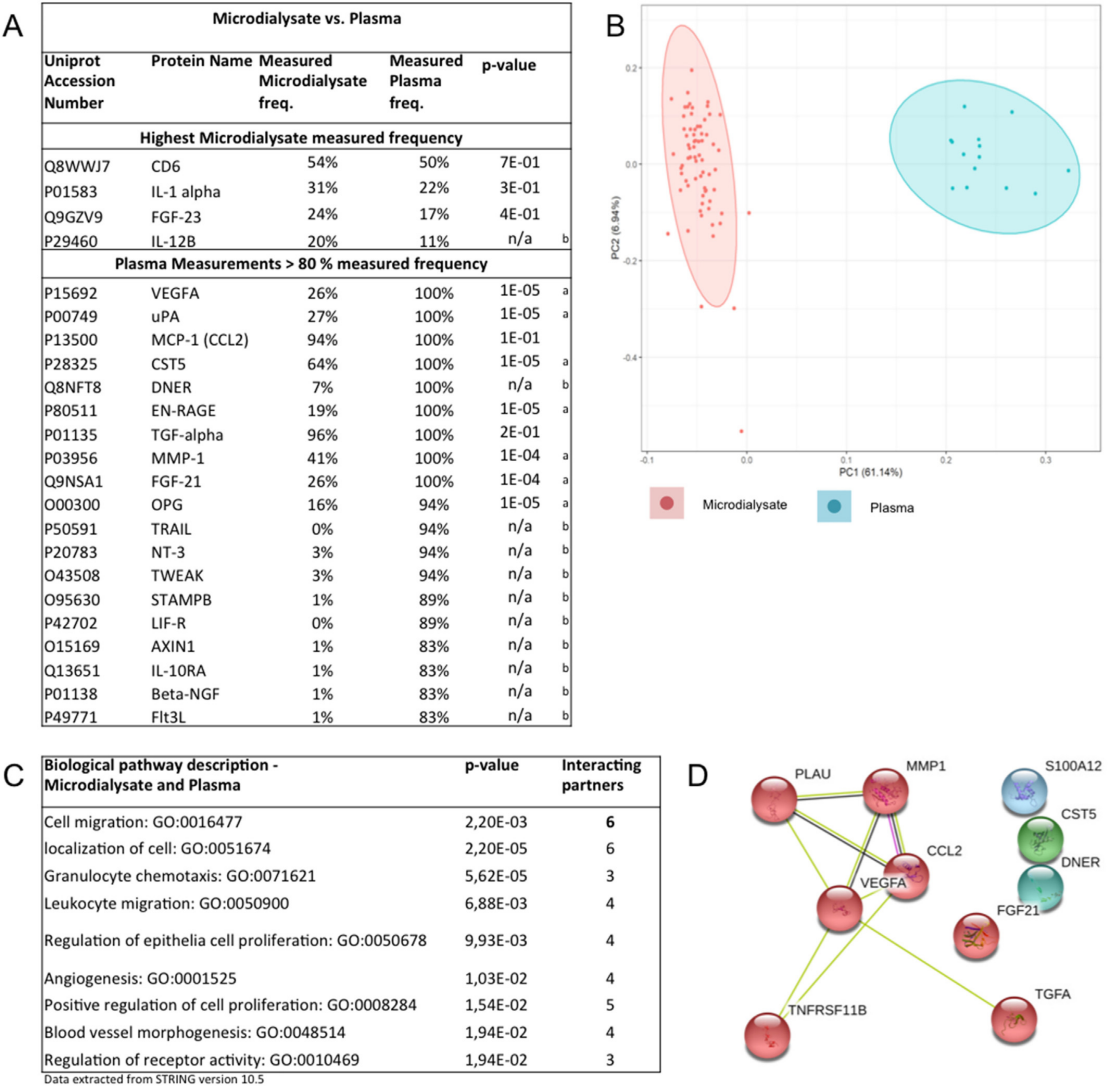
Key proteins of interest from the microdialysates and plasma. (A) Selected proteins of interest based on measured microdialysate freq. or measured plasma freq. (a) One-way ANOVA comparison between microdialysate and plasma. *p*-values <.05 were considered significant. (b) Not available information (n/a). (B) Principal component analysis (PCA) of microdialysate and plasma samples. Analysis was based on NPXs of all proteins for each individual samples (smaller dots). The ellipses indicate the percentage of variability by PC1 and PC2 using all individual samples for a given group. The colored ellipses denote 95% confidence intervals for the different groups. Each axis is labeled with the percent total variance. The two labels correspond to microdialysates (red) and plasma (blue). (C) Biological interaction of proteins measured in both microdialysates and plasma. Each description directs to a GO enrichment search *p*-value, involved partners, and GO terms. (D) Graphic representation of proteins identified to have a connection within both groups. The same coloring indicates stronger relation. Data are extracted from STRING version 10.5 (String-db.org). Protein and gene abbreviation of proteins with *p*-values <.05; T-cell surface glycoprotein Cd6 isoform, CD6; interleukin-1 alpha, IL-1 alpha; fibroblast growth factor 23, FGF-23; vascular endothelia growth factor A, VEGF-A; urokinase-type plasminogen activator, uPA (PLAU); monocyte chemotactic protein 1, MCP-1; cystatin D, CST5; delta and notch-like epidermal growth factor-related receptor, DNER; protein S100-A12, EN-RAGE (S100A12); transforming growth factor alpha, TGF-alpha (TGFA); matrix metalloproteinase-1, MMP-1; fibroblast growth factor 21, FGF-21; osteoprotegrin, OPG (TNFRSF11B).

Data below LOD was not included in the analysis. The explorative cut-off was used to locate the different protein measured frequency across sample conditions. 19 plasma proteins exceeded the explorative cut-off of data measured frequency > 80%, and 4 proteins in microdialysates displayed higher measured frequency than that of plasma (Fig. 1A). In total, 23 proteins were included for statistical tests.

## 3 Results

### 3.1 The explorative inflammatory profile in all microdialysates and plasma

Out of the 23 investigated proteins, 19 were associated with the highest measured frequency in plasma (Fig. 1A). Four proteins; CD6, Il-1α, FGF-23, and IL-12B had the highest detectability in microdialysates (Fig. 1A). Among the explorative proteins that were excluded because they were placed between 50% < plasma measurements <80% measured frequency were CD6, SCF, CXCL10, CD40, IL5 and CSF-1.

**Fig. 2:**
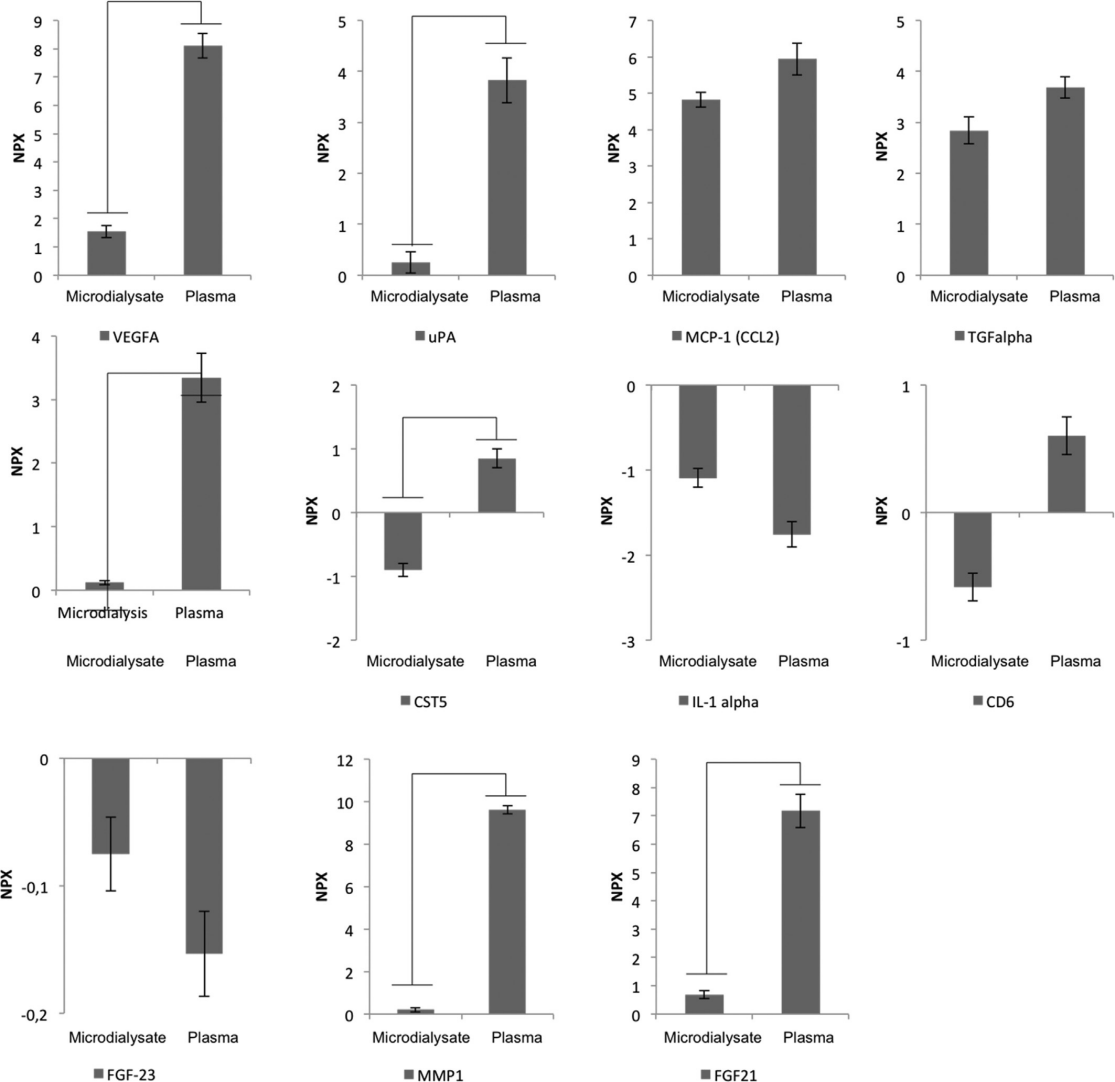
Normalized protein expression (NPX) of specific proteins associated with inflammation. Vertical axes represent arbitrary Log-2 scaled NPX. Horizontal axes represent specific proteins from microdialysate and plasma. *p*-values <.05 are listed in Fig. 1A.

**Fig. 3:**
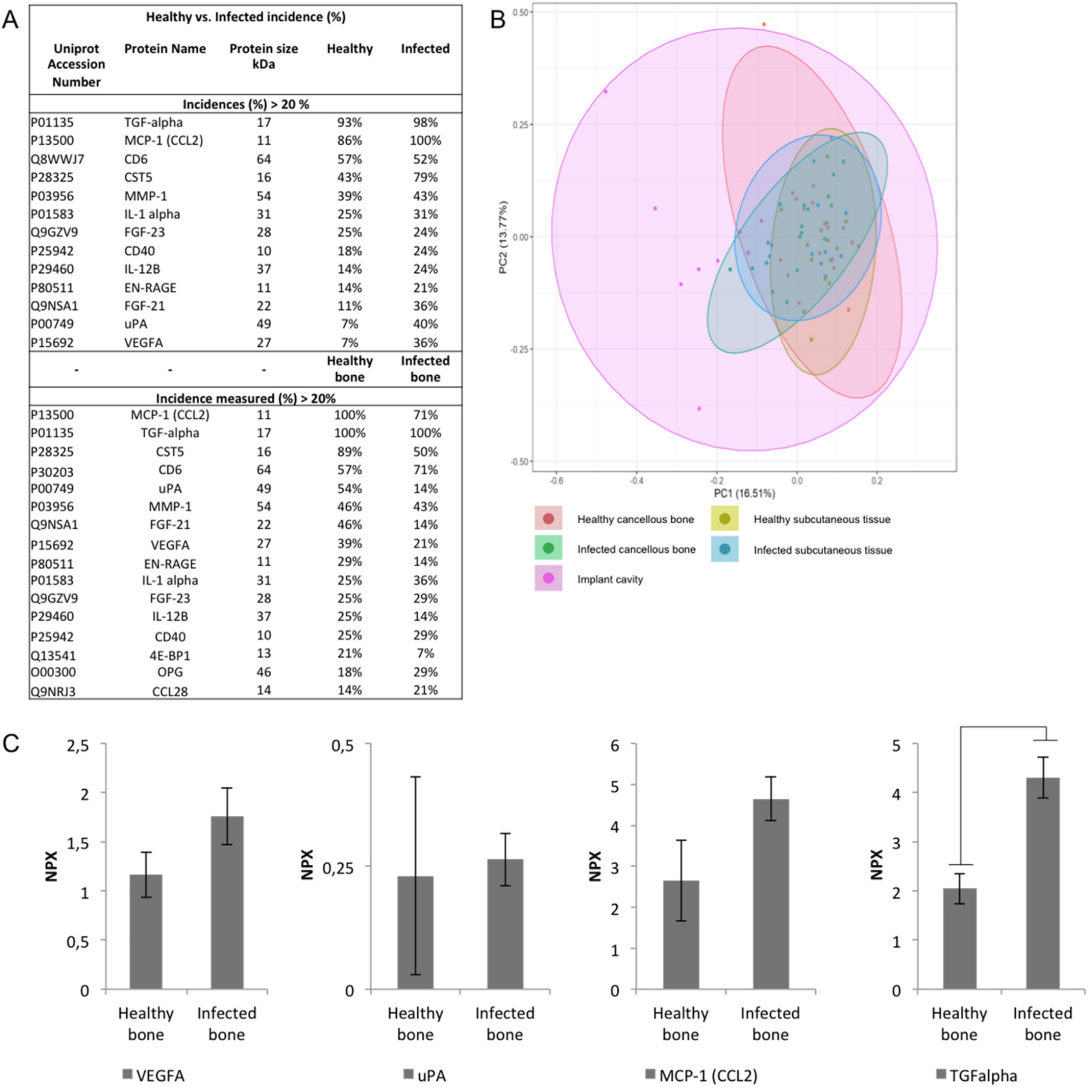
Key proteins of interest for healthy and infected recipients and in bone. (A) Proteins registered with a percentage of incidence >20 percentage for healthy and infected recipients, and healthy and infected bone (B) Principal component analysis (PCA) of the five compartments; healthy cancellous bone, healthy subcutaneous tissue, infected cancellous bone, infected subcutaneous tissue, and the implant cavity. Analysis was based on NPXs of all proteins for each individual sample (smaller dots). The ellipses indicate the percentage of variability by PC1 and PC2 using all individual samples for a given group. The colored ellipses denote 95% confidence intervals for the different groups. Each axis is labeled with the percent total variance. The five labels correspond to healthy cancellous bone (red), healthy subcutaneous tissue (yellow), infected cancellous bone (green), infected subcutaneous tissue (blue), and the implant cavity (pink). (C) NPX of specific proteins associated with angiogenesis for healthy cancellous bone and the infected bone compartments. Vertical axes represent arbitrary Log-2 scaled NPX. Horizontal axes represent specific proteins from Healthy or infected Bone. *p*-values <.05 are considered significant.

The possible associations among the explored proteins were visualized in a principal component object score plot (Fig. 1B). The two groups clustered separately and the groups differed in protein expression levels. Furthermore, there were four outliers placed close to or outside the 95% confidence boundary of the microdialysates. A STRING search was performed to data explore the putative target proteins in plasma. A broad range of gene ontology (GO) terms (biological processes) belonging to the immune response, leukocyte migration (GO:0050900), angiogenesis (GO:0001525), and blood vessel morphogenesis (GO:0048514) were identified but also cell mechanisms were identified based on key proteins of interest (Fig. 1C). Biological interactions between key proteins of interest are visualized in Fig. 1D. Fig. 2 summarize the great dispersal of NPX values between microdialysate and plasma. E.g., TGFα and MCP-1 (CCL2) did not discriminate in NPX indicating the same measurability comparing local versus systemic tissue fluid. CD6, Il-1α, and FGF-23 illustrate the same measurability pattern as TGFα and MCP-1 but all proteins had a molecular weight larger than the microdialysis membrane cut off of 20 kDa (Fig. 2, Fig. 3A). Significant differences were seen for vascular endothelia growth factor A (VEGFA), uPA, OPG, CST5, MMP1, and FGF21 between microdialysates and plasma with the highest NPX expression in plasma (Fig. 2).

### 3.2 The incidence of inflammatory proteins in healthy and infected recipients, and healthy and infected bone

The percentage of incidence for a given inflammatory protein between healthy and infected recipients, and healthy and infected bone indicated similar incidence frequency. However, some inflammatory proteins were more dominant in the infected compartments. The specific bone biomarker, OPG, was present in larger amounts in microdialysate from infected bone compared with healthy bone (Fig. 3A). The same tendency was observed with uPA and VEGFA (Fig. 1A and 3A). Furthermore, we observe a large heterogeneity within the protein size from 11 kDa to 64 kDa (Fig. 3A). We found no significant difference among the five compartments and the variance structure clusters more intricate, as depicted by the PCA plot Fig. 3B.

Among the biological interactions observed in Fig. 1D, angiogenesis displayed with four influencing partners such as VEGFA, urokinase-type plasminogenactivator (PLAU), monocyte chemoattractant protein 1 (CCL2), and transforming growth factor alpha (TGFα). We assessed this interaction in healthy bone and infected bone and observed a tendency towards a higher NPX expression in infected bone but only TGFα demonstrated significance, *p*-value 2.10E-03, Fig. 3C.

## 4 Discussion

In infectious diseases, it is often difficult to anticipate the local concentration of molecules or particular tissue homeostasis, as the plasma level may not reflect the local environment or vice versa. The importance of a specific local immune response in bone infections seems logical as the blood supply is severely impaired during osteomyelitis. Recently, it was demonstrated that infected bone tissue produce a local up-regulation of acute phase proteins during bacterial bone infection with no simultaneous expression of acute phase proteins in liver and serum (Luthje et al., 2020). This explorative porcine study appears to be the first study to investigate the local in vivo inflammatory response by means of microdialysis and PEA in an implant-associated osteomyelitis model. The main finding was that microdialysis was successfully applied for sampling of extracellular fluid followed by assessment of in vivo inflammatory biomarkers by means of PEA. Therefore, microdialysis is a representative method for future in situ investigations of the local inflammatory proteins of complex tissues such as bone tissue.

Surgical and drilling procedures introduce an obligate local inflammatory response. Nevertheless, a tendency of higher NPX in the infected bone tissue in comparison to healthy bone tissue was identified for proteins associated with angiogenesis and bone remodeling: OPG, TGFα, MCP-1, VEGFA, and uPA. OPG is a bone biomarker and TGFα is a known regulator of bone homeostasis balancing formation and resorption of bone, especially on osteoclast stimulation (Loi et al., 2016; Luthje et al., 2018). MCP-1 (CCL2) is produced by macrophages and monocytes and recognized to be involved in inflammation and physiological bone remodeling (Siddiqui and Partridge, 2017). VEGFA and uPA have convergent physiological events including angiogenesis (Breuss and Uhrin, 2012). As both legs were subject to equal surgical and drilling procedures, these differences are likely to be attributed to the bacterial infection. Putting our findings into perspective, it is well known that infection in bone induces intra-trabecular suppuration which results in ischemic osseous sequestration and reduced vascularization (Lew and Waldvogel, 1997). Therefore, it is reasonable to suggest that the trend of higher expression level of angiogenic and bone remodeling factors in infected bone found in the present study, is associated with regulation of tissue homeostasis balancing formation and resorption of bone. As such, our present findings of the inflammatory composition in infected bone tissue acknowledge the current literature of the histopathological changes.

Our findings raise an interesting discussion regarding the origin of the osteogenic- and angiogenic responses after inflammation and how it is controlled. A local infection may retain the inflammatory response by hindering systemic communication, but it may also be vice versa. Although the presence of suppuration in bone may be the primary cause of decreased antibiotic penetration and insufficient oxygen supply (Tottrup et al., 2016; Jensen et al., 2017b; Bue et al., 2018a), local angiogenic factors may be able to reach the systemic circulation. The systemic circulation transports molecular substances to the local site of inflammation, and in bone these substances pass from the capillary walls into the interstitial fluid space of bone via fluid flux movement (Piekarski and Munro, 1977; Cowin and Cardoso, 2015). We found that some of the proteins, e.g. TGFα and MCP-1, were expressed equally locally and systemically, others e.g. uPA and VEGFA exhibited higher measurability in plasma, whereas e.g. CD6 and Il-1α displayed the highest detectability in the microdialysates. We demonstrate parallel detectability of the systemic range of cytokines and chemokines as from the investigated local tissue compartments, indicating the same occurrence of molecules in the local environment as within plasma. However, the implant is less tightly clustered than the other compartments, which coincide with the low antibiotic penetration found in the implant cavity, suggesting an environment of low penetration (Tottrup et al., 2016; Jensen et al., 2017b; Bue et al., 2018a). From a future perspective, it could be speculated that some specific inflammatory proteins convey to sole local comumnication, whereas other systemic inflammatory biomarkers may be able to reflect the local environment in bone. Ultimately, this may allow for different monitoring approaches of the infection progression within bone tissue.

The present study has a number of appreciable limitations. Importantly, the microdialysate concentrations only allow for an explorative comparison to the absolute plasma concentrations. Due to a perfusion rate of the microdialysis system of 1 μL/min, the protein relative recovery was not 100% in this study setup, and f recovery was not estimated. Therefore, the microdialysate results are underestimated in the investigated local tissue compartments compared to plasma and can only safely be regarded as representative of the changes in the concentrations ratios and the variation between compartments. Furthermore, the membrane cut-off of 20 kDa excludes approximately two thirds of the key protein from the Olink Inflammatory Panel. However, we detected some molecules >20 kDa, suggesting an interstitial environment that bypasses the 20 kDa microdialysis membrane. There may be several explanations to this, e.g. a high content of local protein concentrations and locally degraded proteins. Furthermore, the 20 kDa membrane cut-off is only an approximation. For future in vivo microdialysis studies assessing inflammatory markers in infected tissues, the application of a higher cut-off membrane, a lower perfusion flow rate, equal membrane lengths, different sampling intervals and introduction of a systemic (intravenous) microdialysis catheter should be considered. Finally, we applied a human PEA on porcine fluids. It is difficult to evaluate the extent of this limitation, since the exact interspecies differences of the investigated 92 inflammation-related protein biomarkers are unknown. This may have resulted in unfitting PEA antibody probes. Given the reproducibility of this porcine osteomyelitis model, future development of coherent porcine PEAs will significantly contribute in the understanding of the complex interplay of in vivo inflammatory processes in bone infections.

## 5 Conclusion

In conclusion, this explorative study resulted in the finding of an angiogenic and ostogenic inflammatory protein composition within infected bone, in support of the current histopathological knowledge. Microdialysis may represent a valid method for sampling of material for protein investigation of the in vivo inflammatory composition within the extracellular environment in a rather complex tissue such as infected bone tissue. To what extent the specific inflammatory protein composition in bone tissue is involved in the pathological changes of osteomyelitis, in the local and systemic inflammatory development, and also the following regenerative response needs further investigation.

The following are the supplementary data related to this article.

Supplementary Fig. 1 List of the proteins analyzed using PEA – inflammatory panel from Olink. Supplementary Fig. 1

## Transparency document

Transparency document Image 1

## CRediT authorship contribution statement

**Mats Bue:**Conceptualization, Data curation, Formal analysis, Investigation, Writing - original draft, Writing - review & editing.**Natasja Leth Bergholt:**Conceptualization, Formal analysis, Investigation, Writing - original draft, Writing - review & editing.**Louise Kruse Jensen:**Conceptualization, Data curation, Investigation, Writing - review & editing.**Henrik Elvang Jensen:**Conceptualization, Investigation, Writing - review & editing.**Kjeld Søballe:**Conceptualization, Investigation, Writing - review & editing.**Maiken Stilling:**Conceptualization, Investigation, Writing - review & editing.**Pelle Hanberg:**Conceptualization, Data curation, Formal analysis, Investigation, Writing - review & editing.

## Declaration of competing interest

The authors declare that they have no known competing financial interests or personal relationships that could have appeared to influence the work reported in this paper.
