# Supplementary material for: Inflammatory proteins in infected bone tissue – An explorative porcine study
Source: Bone Rep. 2020 Jun 26;13:100292. doi: 10.1016/j.bonr.2020.100292 (PMC7330156; doi:10.1016/j.bonr.2020.100292)
Supplement: Supplementary Fig. 1 — List of the proteins analyzed using PEA – inflammatory panel from Olink. [file mmc1.docx]

**Supplementary**

Supplementary Figure 1. List of the proteins analyzed using PEA – inflammatory panel from Olink.
